# Antibiotic Resistance of *Streptococcus pneumoniae* in the Nasopharynx of Healthy Children Less than Five Years Old after the Generalization of Pneumococcal Vaccination in Marrakesh, Morocco

**DOI:** 10.3390/antibiotics12030442

**Published:** 2023-02-23

**Authors:** Sara Amari, Karima Warda, Majda Bouraddane, Mostafa Katfy, Youssef Elkamouni, Lamiae Arsalane, Khalid Zerouali, Said Zouhair, Mohamed Bouskraoui

**Affiliations:** 1 Laboratoire de Lutte Contre les Maladies Infectieuses, Faculté de Médecine et de Pharmacie, Université Cadi Ayyad, Marrakech 40000, Morocco; 2 Service de Microbiologie, CHU Ibn Rochd, Casablanca 20000, Morocco; 3 Laboratoire de Bactériologie, Virologie, et Biologie Moléculaire, Hôpital Militaire Avicenne, Marrakech 40000, Morocco; 4 Service de Pédiatrie, Hôpital Universitaire Mohammed VI, Marrakech 40000, Morocco

**Keywords:** *Streptococcus pneumoniae*, antibiotics, resistance, PSDP, nasopharynx, children, healthy

## Abstract

*Streptococcus pneumoniae* (*S. pneumoniae*) remains one of the most important pathogens causing childhood infections. The spread of antibiotic-resistant bacteria is a leading cause of treatment failure in children. The purpose of this investigation is to report the antibiotic and multidrug resistance (MDR) of *S. pneumoniae* strains isolated from healthy children throughout the years 2020–2022. Antimicrobial susceptibility testing of *S. pneumoniae* strains in selected antimicrobials was performed using disk diffusion and E-test methods on bloodMueller–Hinton agar. The antimicrobials tested included oxacillin, amoxicillin, ceftriaxone, norfloxacin, gentamicin, vancomycin, erythromycin, clindamycin, pristinamycin, tetracycline, chloramphenicol, and trimethoprim-sulfamethoxazole. A total of 201 *S. pneumoniae* strains were isolated from the nasopharynx of healthy children in Marrakesh, Morocco. The highest rate of resistance of *S. pneumoniae* was found in penicillin (57.2%), followed by tetracycline (20.9%), and erythromycin (17.9%). The rates of resistance to clindamycin, trimethoprim-sulfamethoxazole, and chloramphenicol were 14.9%, 4%, and 1.5%, respectively. All isolates were susceptible to norfloxacin, gentamicin, vancomycin, and pristinamycin. Approximately 17% of all *S. pneumoniae* strains were resistant to at least three different antibiotic families. This study showed a low rate of antibiotics resistance among nasopharyngeal *S. pneumoniae* strains, and it is thus essential to monitor *S. pneumoniae* susceptibility in healthy children.

## 1. Introduction

The human nasopharynx is the natural niche of *Streptococcus pneumoniae* [1]. *S. pneumoniae* is a Gram-positive bacterium responsible for a variety of invasive and non-invasive diseases. In addition to this, it also constitutes a leading cause of morbidity and mortality, especially among children younger than 5 years of age [2,3].Generally, children carrying *S. pneumoniae* are mostly asymptomatic, but under some circumstances, they can develop some serious infections, such as pneumonia, meningitis, bacteremia, otitis, and sepsis [4,5].

Antibiotics have solved the problem of treating different infectious diseases, but the rapid rise in antibiotic resistance has affected their effectiveness in recent decades, and even more so in recent years [6]. ßeta-lactams (ß-lactams) are first line antibiotics prescribed for the treatment of *S. pneumoniae* diseases [7]. Macrolides, fluoroquinolones, lincosamides, tetracyclines, and vancomycinare prescribed in cases of ß-lactams resistance and for individuals reporting ß-lactam allergy [8,9].

The spread of antibiotic resistance is actually known as a serious health issue. An increase in morbidity and mortality rates was observed due to pneumococcal disease caused by multidrug-resistant *S. pneumoniae* [10]. Excessive use and misuse of antibiotics are two factors that promote the increase in resistance rate and the spread of MDR bacterial isolates. *S. pneumoniae* is one of the Gram-positive-resistant bacteria responsible for a wide variety of severe infections [11,12]. MDR strains of *S. pneumoniae* have been reported in all parts of the world [13].Penicillin non-susceptible pneumococcus (PNSP) is one of the most frequent profiles of resistance among *S. pneumoniae* isolates. Since the first report in 1960s, PNSP strains have become common across the world [14]. In addition, PNSP strains are mostly known to be associated with other antimicrobial agents (e.g., macrolides, lincosamides, tetracyclines).

To the best of our knowledge, there are little data concerning *S. pneumoniae* antibiotic non-susceptibility in carriage after the introduction of pneumococcal conjugate vaccine 10-valent (PCV10). In Morocco, PCV10 (1, 4, 5, 6B, 7F, 9V, 14, 19F, 18C, and 23F) was introduced into the national immunization program (NIP) in July 2012 for all children.

In the present study, we aim to determine the antibiotic resistance and MDR profiles of *S. pneumoniae* strains isolated from healthy children in Marrakesh, Morocco.

## 2. Results

### 2.1. Characteristics of Study Population

The general characteristics of the children population are presentedin Table 1. In total, 645 nasopharyngeal swabs were collected from healthy children aged between 6 and 60 months. A higher proportion of females were recruited (54.7%; 350/645) with a sex ratio of 1.19. The median age of the included children was 18 months (interquartile range (IQR): 12.0–33.2). The portion of the recruited children who had received an antibiotic treatment during the last three months was 31% (200/645). Nearly half of the included children (49.8%; 321/645) were fully vaccinated by PCV10. *S. pneumoniae* colonization of the nasopharynx was found in 239 (37.1%) of the 645 healthy children.

### 2.2. Antimicrobial Susceptibility Testing

The antimicrobial susceptibility of 201 *S. pneumoniae* was tested against eleven antibiotics (twenty-eight *S. pneumoniae* strains were non-viable after conservation at −80 °C). The overall resistance rate to different antibiotics was as follows: 57.2% (115/201) to oxacillin; 20.9% (42/201) to tetracycline; 17.9% (36/201) to erythromycin; 14.9% (30/201) to clindamycin; 11% (22/201) to pristinamycin; 4% (8/201) to trimethoprim-sulfamethoxazole; and 1.5% (3/201) to chloramphenicol. All isolates were susceptible to norfloxacin, gentamicin, and vancomycin. More details regarding the non-susceptibility rates of the *S. pneumoniae* isolates are listed in Table 2.

Concerning the oxacillin-positive isolates, the rate of resistance to amoxicillin (oral administration, MIC>1) was 21.4%. Among amoxicillin-resistant strains, 14.3% were highly resistant, with MIC values in the range ≤3–8 mg/L. However, the rate of ceftriaxone intermediate resistance was 14.3%. None of the oxacillin-positive isolates were resistant to ceftriaxone (indications other than meningitis, MIC >2 mg/L). The macrolides-resistant phenotypes are presented in Table 3. The MLS_B_ phenotype (co-resistance to erythromycin and clindamycin) was reported in 22/201 (10.9%) of the *S. pneumoniae* strains, while 13/34 (38.2%) were MLS_B_ constitutive (negative D-test) and 9/34 (26.4%) were MLS_B_ inducible (positive D-test). The M phenotype (resistance only to erythromycin) was reported in 12/201 (6%) of the strains.

### 2.3. Multidrug Resistance among S. pneumoniae Isolates

The antibiotic resistance profiles of the *S. pneumoniae* isolates are presented in Table 4. Oxacillin, amoxicillin, and ceftriaxone were classified as ß-lactams. In contrast, erythromycin, clindamycin, tetracycline, chloramphenicol, and trimethoprim-sulfamethoxazole were classified as macrolides, lincosamides, tetracyclines, phenicols, and a folate pathway inhibitor, respectively.

MDR was defined as resistant to at least two different families of antimicrobials. The rate of MDR was 17% (34/201). The most common MDR profile was ß-lactams, macrolides, lincosamides, streptogramins, and tetracyclines (8.5%; 17/34). MDR among oxacillin-positive strains were mostly associated with non-susceptibility to macrolides (25.9% compared to3.4% of oxacillin negative *S. pneumonia* strains).

### 2.4. Resistance Profiles of Oxacillin-Positive Strains to Antibiotics

Statistical analyses showed that the rates of non-susceptibility of oxacillin-positive isolates, compared to other antibiotics, were higher than those observed in susceptible strains. In fact, a statistical difference in non-susceptibility to erythromycin, clindamycin, pristinamycin, and trimethoprim-sulfamethoxazole was found between oxacillin-negative and oxacillin-positive strains (*p* < 0.05). The non-susceptibility rates of oxacillin-positive and oxacillin-negative strains to different antibiotics tested are reported in Table 5.

### 2.5. Serotype Distribution

**Figure 1:**
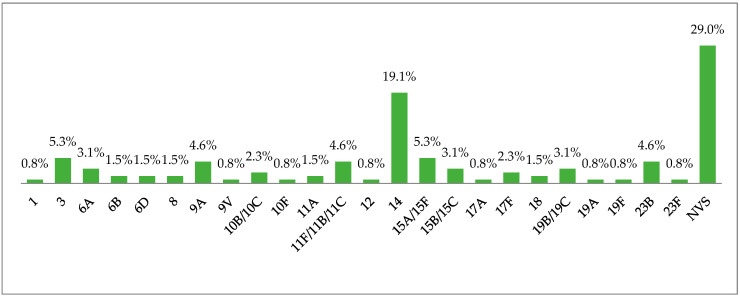
Serotype distribution of *S. pneumoniae* strains isolated from the healthy children’s nasopharynx in Marrakesh, Morocco.

A total of 24 distinct serogroups/types were found among 131 *S. pneumoniae* isolates. Serotypes 14 (*n* =25; 19.1%), 3 (*n* =7; 5.3%), 15A/15F (*n* =7; 5.3%), 9A (*n* =6; 4.6%), 11F/11B/11C (*n* =6; 4.6%), and 23B (*n* =6; 4.6%) were commonly isolated, covering approximatively 43.5% of all strains. A total of 18 strains were non-typeable as they showed no agglutination, but the PCR cpsA reaction was positive. Based on the serotypes contained in the vaccines, the coverage rates of PCV10 and PCV13 were 25.2% (33/149) and 34.4% (45/149), respectively. In addition, non-vaccine serotypes (NVS) constituted 65.6% (86/149). The distribution of the serotypes is shown in Figure 1.

**Figure 2:**
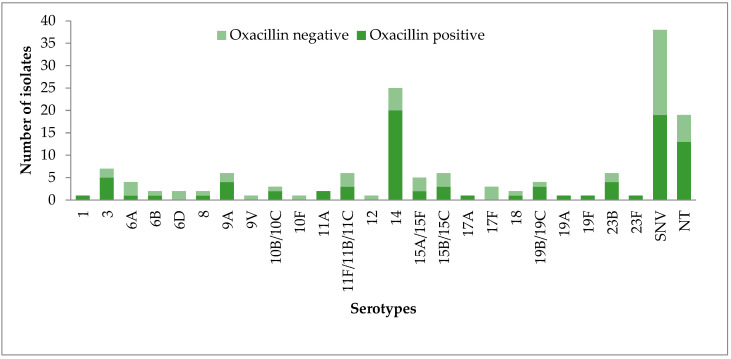
Serotype distribution of *S. pneumoniae* strains according to oxacillin susceptibility.

Among the 131 *S. pneumoniae* isolates serotyped, 76 (58%) were oxacillin-positive. Serotype 14 (26.3%) was the most common serotype that was oxacillin-positive. All *S. pneumoniae* strains detected as serotypes 1, 17A, 19F, and 23F were oxacillin-positive. In addition, the rate of oxacillin-positive isolates among NVS was important (57.9%). More details regarding the capsular serotypes associated with an oxacillin-positive profile are indicated in Figure 2.

In general, serotypes 1, 3, 9A, 10B/10C, 14, 15A/15F, 19F, 19B/19C, and 23B were found to be resistant to at least one antimicrobial. In contrast, serotypes 6A, 6B, 9V, 18, 19A, and 23F were found to be susceptible to all antimicrobials. The highest resistance rate to nearly all antimicrobials was observed in serotype 19F. More details regarding the distribution of *S. pneumoniae* serotypes according to antimicrobials resistance are presented in Table 6.

## 3. Discussion

The study describes the resistance rate of *S. pneumoniae* strains isolated from healthy children in Marrakesh, Morocco. In our country, as in the majority of regional countries, antibiotics are easily obtained without prescription from pharmacies. Incorrect use of antibiotics can potentially promote rates of MDR in children and make treatment of *S. pneumoniae* infections more difficult.

An oxacillin disk (1ug) is usually used to determine *S. pneumoniae* isolates with decreased susceptibility to penicillin (PNSP) [15]. The rate of oxacillin-positive *S. pneumoniae* strains isolated from healthy children was 57.2%. This rate was comparable to the rate of PNSP found in Indonesia (40%) [16] and Belgium (17.7%) [17]. In contrast, it was lower than that found in Brazil (71.4%) [18]. In addition, our results showed that oxacillin-positive strains were associated with resistance to amoxicillin and ceftriaxone, in concordance with the findings reported in France by C. Plainvert et al. [19]. PNSP screening in carriage is of particular interest because of the rapid spread of PNSP strains worldwide [20]. Furthermore, penicillin, and cephalosporins are the preferred treatment for pneumococcal diseases.

Erythromycin belongs to the macrolides class of drugs and is classified as an alternative to penicillin for the treatment of pneumococcal diseases [21]. The main mechanism of resistance to macrolides in *S. pneumoniae* is due to ribosomal methylation, mediated by erm(B) [22] or efflux pumps by mef(E)/mel(msr(D)) [23]. Erythromycin resistance has been recorded as the most prevalent form of antibiotic resistance around the world in recent years [22]. In this study, 17.9% of *S. pneumoniae* were non-susceptible to erythromycin. This rate of resistance to erythromycin remained low compared to other studies conducted in Thailand (18.4%), Cyprus (27.5%), Egypt (40%), and Indonesia (87%), where erythromycin is probably frequently used as treatment [24,25,26,27].

On the other hand, the predominant MLS_B_-constitutive phenotype was observed in 38.2% of the strains tested, followed by the MLS_B_-inducible phenotype, which was detected in 26.4% of pneumococcal strains. Only 6% of the strains exhibited the M phenotype. A study conducted in North Lebanon showed that the MLS_B_-constitutive phenotype (68.9%) was the most frequent phenotype in erythromycin-resistant pneumococci [28]. In line with this, another study performed in Iran revealed that the MLS_B_-constitutive phenotype was observed in 84% of the isolates [29].

Fluoroquinolones are the second alternative used for the treatment of respiratory diseases. In our study, all pneumococcal isolates were fully susceptible to fluoroquinolones. However, recent work examining *S. pneumoniae* isolates collected from different sites in Jordan showed an interesting rate of fluoroquinolone non-susceptibility (83.8%) [30]. Similarly, other studies reported the spread of resistance to fluoroquinolones in the United States and Korea [31,32]. This high rate of susceptibility in our study could be due to the fact that the study included healthy young children (6 to 60 months).

MDR remains a growing global issue in both developed and developing countries. The overuse of antimicrobial agents is a major contributor to the emergence of MDR pneumococci. The increase in the rate of MDR *S. pneumoniae* strains could have several impacts, such as higher medical costs, treatment failure [22], and increased mortality [11]. It is known that the nasopharyngeal carriage of *S. pneumoniae* in children increases the risk of pneumococcal diseases and the spread of antimicrobial-resistant *S. pneumoniae*. In this study, MDR was mostly detected among oxacillin-positive isolates compared to oxacillin-negative isolates. The majority of oxacillin-positive isolates are typically resistant to other class of antibiotics, such as macrolides and tetracyclines. In the present study, the rate of MDR *S. pneumoniae* strains was 17%.This rate of MDR was lower than other rates reported in published studies. MDR was found with rates of 31.6% in Thailand [26], 33.3% in Ethiopia [33], 46.1% in China [34], and 80% in Vietnam [35].The rate of *S. pneumoniae* resistant isolates has increased worldwide [11]. However, the antimicrobial susceptibility testing carried out in this current study showed low levels of resistance, as previously reported in a Moroccan study conducted among children with invasive diseases [36]. This downward trend in resistance was observed for tetracycline, erythromycin, clindamycin, trimethoprim-sulfamethoxazole, and chloramphenicol. This finding suggests that PCV10 reduces antibiotic resistance among children.

Furthermore, our study showed that non-vaccine serotypes were found to be frequent in carriage. This result was in agreement with earlier studies in countries that have introduced pneumococcal vaccinations [24,37,38]. 9A, 11F/11B/11C, 15A/15F, and 23B were the most frequent non-vaccine serotypes detected in healthy children. In Cyprus, the non-vaccine serotypes detected in a study that included 1105 healthy children, aged between 6 and 36 months, were 15A, 6C, 23B, and 15B [39]. Among Bangladeshi children, 34, 15B, 17F, and 35B were the predominant non-vaccine serotypes, accounting for 43.6% [40]. The distribution of pneumococcal non-vaccine serotypes varies across studies due to many factors, such as age, country, study period, and time of vaccine introduction.

Concerning the distribution of *S. pneumoniae* serotypes according to antimicrobial resistance, serotype 14 was found to be the most common oxacillin-positive serotype, consistent with results from a study conducted by Yahiaoui et al. [41]. Similarly, a study conducted in Russia among children revealed that the highest PNSP rate was observed among serotypes 14, 23F, 6B, 10A, and 19F [42]. In addition, it is important to note that serotype 19A was less common in our study (one oxacillin-positive isolate susceptible to all other antibiotics). However the emergence of serotype 19A with a high level of resistance was reported in the PCV10 era in Brazil [43].

This study has limitations. We only included healthy children in Marrakesh, which may affect the representativeness of the entire Moroccan child population. Therefore, a national study from more public health centers is recommended.

## 4. Methods

### 4.1. Study Design and Population

This prospective study was conducted in public health centers in Marrakesh, Morocco. Healthy asymptomatic children, aged less than five years old, visiting public health centers for vaccinations were randomly selected. A healthy child was defined as a child presenting with no fever, no signs of respiratory infections, and no antibiotic consumption during the last seven days. A questionnaire containing demographic, socio-economic, and clinical data was completed. The collection of nasopharyngeal specimens was performed using a sterilized flocked nylon swab (COPAN swab collection, 482CE), placed in a medium containing skim-milk tryptone glucose glycerol. One nasopharyngeal swab was collected for each child. Swabs were sent to the Microbiology-Virology Laboratory of Faculty of Medicine and Pharmacy in Marrakesh, Morocco. The study period spanned the years 2020–2022. Only children with a *S. pneumoniae*-positive culture were recruited in this study.

### 4.2. Ethical Permission

The Ethics Committee of the University Hospital Center of Mohammed VI in Marrakesh, Morocco approved this study (Reference number 26/2022). Written informed consent was obtained and signed by the parents or legal guardians of each child before collecting nasopharyngeal specimens. The study was performed anonymously.

### 4.3. Identification of S. pneumoniae Isolates

Nasopharyngeal specimens were initially cultured on colistin nalidixic acid agar (Biolife, Milano, Italia), supplemented with 5% blood and incubated overnight at 37 °C in a 5% CO_2_ atmosphere. *S. pneumoniae* isolates were identified based on typical colony morphology (dark green colonies with depressed centers), alpha hemolysis, Gram-positive staining, negative catalase reaction, optochin susceptibility, bile solubility, and an agglutination test Slidexpneumo-Kit (Bio Mérieux, Craponne, France). *S. pneumoniae* colonies were then transferred into brain heart infusion broth (Biokar, Allone, France), supplemented with 15% glycerol and kept at −80 °C until use.

### 4.4. Antimicrobial Susceptibility Testing

Antimicrobial susceptibility was tested on Mueller–Hinton agar (Biokar, Allone, France), supplemented with 5% sheep blood using the disk diffusion method (Kirby-Bauer). ß-lactams resistance in *S. pneumoniae* was firstly determined using an oxacillin disk (OXA; 1 μg), according to EUCAST (2022) recommendations. Instead, for strains with an oxacillin zone diameter <20mm, minimum inhibitory concentrations (MICs) of amoxicillin and ceftriaxone were tested.The MICs were checked using E-teststrips (Bio Mérieux, Craponne, France), graduated from 0.016 to 256 mg/L.The *S. pneumoniae* isolates were considered sensitive, intermediate, and resistant with the following reading: ≤1 mg/L, 1.5–2 mg/L, and >2 mg/L, respectively, for amoxicillin; and ≤0.5 mg/L, 0.75–2 mg/L, and >2 mg/L, respectively, for ceftriaxone. Antimicrobial susceptibility was also tested against norfloxacin (NOR; 10 μg), gentamicin (GEN; 500 μg), vancomycin (VAN; 5 μg), erythromycin (ERY; 15 μg), clindamycin (CLN; 2 μg), pristinamycin (PTN; 15 μg), tetracycline (TET; 30 μg), chloramphenicol (CHL; 30 μg), and trimethoprim-sulfamethoxazole (SXT; 1.25/23.75 μg). In 5% CO_2_, plates were incubated for 18–24 h at 37 °C.

In case the *S. pneumoniae* strain was resistant to erythromycin, D-testing was performed to detect the following phenotypes: MLS_B_-inducible phenotype; MLS_B_-constitutive phenotype; and M phenotype. On Mueller–Hinton agar (Biokar, Allone, France) supplemented with 5% sheep blood, an erythromycin disk (15 μg) was placed 12 mm away from a clindamycin disk (2 μg) and incubated overnight for 20–24 h. A positive D-test means a flattened zone of the clindamycin disk was observed.

### 4.5. Capsular Typing

The detection of *S. pneumoniae* serogroups was performed using the IMMULEX PNEUMOTEST agglutination test (Staten Serum Institut, Copenhagen, Denmark). The serotyping was performed usingreal-time polymerase chain reaction (RT-PCR) following the recommendations published by the Centers for Disease Control and Prevention (CDC). Quellung reaction was accomplished for serotyping serogroups 9, 6, and 23.

### 4.6. Statistical Analyses

Data were entered and analyzed using the SPSS/PC 23.0 program (SPSS Inc., Chicago, IL, USA).Participant characteristics were expressed by counts and percentages, or median and interquartile range. The χ^2^ test was done to compare the non-susceptibility of oxacillin-positive and oxacillin-negative strains to other antibiotics. A *p*-value under 0.05 (*p* ≤0.05) was considered statistically significant.

## 5. Conclusions

This study presents epidemiological data on the resistance of nasopharyngeal strains of *S. pneumoniae* isolated from healthy children in Marrakesh, Morocco. Our results show a low carriage of resistant MDR strains to antibiotics frequently used in the treatment of pneumococcal infections and a decrease in the rate of PCV10 vaccine, alongside an increase in non-vaccine serotypes after the widespread use of PCV10.Therefore, it is necessary to act on the parameters that maintain this low rate of resistance, namely self-medication of the population and the irrational use of antibiotics. For this, the establishment of an appropriate law to control the non-regulatory sale of antibiotics without a prescription from pharmacists is strongly recommended. The development of a permanent awareness program for private practitioners, the prescription of antibiotics, and the implementation of a hospital antibiotic stewardship program are also recommended. It is also necessary to promote the role of the National Pneumococcal Observatory as a federator in the continuous surveillance of antibiotic resistance to *S. pneumoniae* from carriage and clinical isolates.

## Figures and Tables

**Table 1:** Characteristics of study participants.

Characteristic of Children	Study Population	Children with Carriage of *S. pneumoniae*
Total, *n* (%)	645 (100)	239 (37.1)
Gender		
Male, *n* (%)	295 (45.7)	132 (44.7)
Female, *n* (%)	350 (54.7)	107 (30.6)
Age in months, median (IQR)	18 (21.5)	18 (23)
Antibiotic treatment ≤3 months, *n* (%)	200 (31)	57 (28.5)
Fully vaccinated, *n* (%)	321 (49.8)	112 (34.9)

*n* = total number; %: percentage; IQR: interquartile range.

**Table 2:** Antimicrobial susceptibility of *S. pneumoniae* strains isolated from the healthy children’s nasopharynx in Marrakesh, Morocco.

Type of ATB	Disk Content	Breakpoints EUCAST	Total Number of the Isolates	S (N)	S (%)	R (N)	R (%)
OXA	1 μg	≤20–>20 mm	201	86	42.8	115	57.2
AMX	-	MIC ≤ 1–2 mg/L	115	90	78.6	24	21.4
CFR	-	MIC ≤ 0.5–2 mg/L	115	99	85.7	16	14.3
NOR	10 μg	≤10–>10 mm	201	201	100	-	-
GEN	500 μg	≤17–>17 mm	201	201	100	-	-
VAN	5 μg	≤16–>16 mm	201	201	100	-	-
ERY	15 μg	≤22–>19 mm	201	165	82.1	36	17.9
CLN	2 μg	≤19–>19 mm	201	171	85.1	30	14.9
PTN	15 μg	≤19–>19 mm	201	179	89	22	11
TET	30 μg	≤25–>25 mm	201	159	79.1	42	20.9
CHL	30 μg	≤21–>21 mm	201	198	98.5	3	1.5
SXT	1.25/23.75 μg	≤13–>10 mm	201	193	96	8	4

ATB: antibiotic; N: total number; S: susceptible; R: resistant (intermediate + resistant); %: percentage; OXA: oxacillin; AMX: amoxicillin; CFR: ceftriaxone; NOR: norfloxacin; GEN: gentamicin; VAN: vancomycin; ERY: erythromycin; CLN: clindamycin; PTN: pristinamycin; TET: tetracycline; CHL: chloramphenicol; SXT: trimethoprim-sulfamethoxazole.

**Table 3:** Macrolides-resistant phenotypes of *S. pneumoniae* strains isolated from the healthy children’s nasopharynx in Marrakesh, Morocco.

	Erythromycin-Resistant Strains (N=34)	
	N	%
Clindamycin-resistant strains	22	64.7
D-test positive	9	26.4
D-test negative	13	38.2
Clindamycin-susceptible strains	12	35.9
Pristinamycin-resistant strains	22	64.7
Pristinamycin-susceptible strains	12	35.9

**Table 4:** Antibiotic resistance profile of *S. pneumoniae* strains isolated from the healthy children’s nasopharynx in Marrakesh, Morocco.

Profile of Resistance		*S. pneumoniae* Isolates	
		N	%
Coresistance	ß-lactams, Macrolides	3	1.5
	ß-lactams, Lincosamides	2	1
	ß-lactams, Tetracyclines	6	3
	Macrolides, Tetracyclines	2	1
	ß-lactams, Folate pathway inhibitor	1	0.5
Multiresistance	ß-lactams, Macrolides, Tetracyclines	5	2.5
	ß-lactams, Macrolides, Lincosamides	1	0.5
	Macrolides, Lincosamides, Tetracyclines	1	0.5
	ß-lactams, Tetracyclines, Folate pathway inhibitor	2	1
	ß-lactams, Macrolides, Lincosamides, Tetracyclines	3	1.5
	ß-lactams, Macrolides, Lincosamides, Streptogamines	3	1.5
	ß-lactams, Macrolides, Lincosamides, Streptogamines, Tetracyclines	17	8.5
	Macrolides, Lincosamides, Streptogamines, Phenicols	1	0.5
	ß-lactams, Macrolides, Lincosamides, Streptogamines, Phenicols	1	0.5

N: total number; %: percentage.

**Table 5:** Non-susceptibility rates of oxacillin-positive and oxacillin-negative strains to other antibiotics.

	Oxacillin-Positive	Oxacillin-Negative	*X* ^2^	*p*-Value
ERY	31 (86.1%)	5 (13.9%)	15.213	<0.001
CLN	26 (86.7%)	4 (13.3%)	12.706	<0.001
PTN	19 (95.4%)	3 (4.6%)	8.723	<0.05
TET	32 (76.2%)	10 (23.8%)	8.022	<0.05
CHL	2 (66.6%)	1 (33.4%)	0.118	0.732
SXT	57 (75%)	19 (25%)	1.109	<0.05

**Table 6:** Distribution of *S. pneumoniae* serotypes according to antimicrobial resistance.

Capsular Serotypes	Total (N)	Antimicrobials									
		Erythromycin		Lincomycin		Tetracycline		Chloramphenicol		SXT	
PCV10											
1	1	1	100%	0	0%	0	0%	0	0%	0	0%
6B	2	0	0%	0	0%	0	0%	0	0%	0	0%
9V	1	0	0%	0	0%	0	0%	0	0%	0	0%
14	25	1	4%	1	4%	4	16%	0	0%	3	12%
18	2	0	0%	0	0%	0	0%	0	0%	0	0%
19F	1	1	100%	1	100%	1	100%	0	0%	0	0%
23F	1	0	0%	0	0%	0	0%	0	0%	0	0%
PCV13											
3	7	3	43%	1	14%	3	43%	0	0%	0	0%
6A	4	0	0%	0	0%	0	0%	0	0%	0	0%
19A	1	0	0%	0	0%	0	0%	0	0%	0	0%
Non-PCV											
6D	2	0	0%	0	0%	0	0%	0	0%	0	0%
8	2	0	0%	0	0%	0	0%	0	0%	0	0%
9A	6	1	17%	0	0%	1	17%	0	0%	0	0%
10B/10C	3	2	67%	0	0%	2	67%	0	0%	0	0%
10F	1	0	0%	0	0%	0	0%	0	0%	0	0%
11A	2	0	0%	0	0%	0	0%	0	0%	0	0%
11F/11B/11C	6	0	0%	0	0%	0	0%	0	0%	0	0%
12	1	0	0%	0	0%	0	0%	0	0%	0	0%
15A/15F	7	1	14%	0	0%	1	14%	0	0%	0	0%
15B/15C	4	0	0%	0	0%	0	0%	0	0%	0	0%
17A	1	0	0%	0	0%	0	0%	0	0%	0	0%
17F	3	0	0%	0	0%	0	0%	0	0%	0	0%
19B/19C	4	0	0%	1	25%	0	0%	0	0%	2	50%
23B	6	1	17%	0	0%	1	17%	0	0%	0	0%
SNV	38	4	11%	2	5%	7	18%	0	0%	0	0%
NT	18	5	28%	6	33%	2	11%	2	11%	2	11%

## Data Availability

Data related to the current study can be accessed upon reasonable request to ka.warda@uca.ac.ma and sara.amari@edu.uca.ma.
